# Prophylactic antibiotics for manual removal of retained placenta during vaginal birth: a systematic review of observational studies and meta-analysis

**DOI:** 10.1186/s12884-015-0752-4

**Published:** 2015-11-26

**Authors:** Ezinne C. Chibueze, Alexander J. Q. Parsons, Erika Ota, Toshiyuki Swa, Olufemi T. Oladapo, Rintaro Mori

**Affiliations:** Department of Health Policy, National Center for Child Health and Development, 10-1-2 Okura, Setagaya-ku, Tokyo 157-8535 Japan; Department of Global Health Policy, Graduate School of Medicine, University of Tokyo, Tokyo, Japan; Graduate School of Human Sciences, Osaka University, Osaka, Japan; Department of Reproductive Health and Research, including UNDP/UNFPA/UNICEF/WHO/World Bank Special Programme of Research, Development and Research Training in Human Reproduction (HRP), World Health Organization, Geneva, Switzerland

**Keywords:** Antibiotic prophylaxis, Retained placenta, Vaginal birth, Systematic review, Manual removal, Meta-analysis

## Abstract

**Background:**

Manual removal of the placenta is an invasive obstetric procedure commonly used for the management of retained placenta. However, it is unclear whether antibiotic prophylaxis is beneficial in preventing infectious morbidity. We conducted a systematic review to determine the efficacy and safety of routine use of antibiotics for preventing adverse maternal outcomes related to manual placenta removal following vaginal birth.

**Methods:**

A detailed search of MEDLINE, EMBASE, Cochrane library and the CINAHL databases was conducted for non-randomized studies involving women undergoing manual placenta delivery after vaginal births and where antibiotic prophylaxis use was compared with no treatment or placebo to prevent maternal infection. Search terms including ‘delivery, obstetric’, ‘placenta, retained’, ‘anti-infective agents’, and ‘chemoprevention’ were used.

**Results:**

Of the 407 citations that resulted after elimination of duplicates, 81 full texts were potentially eligible after independent assessment of the title and abstracts. Independent review of the full texts identified three eligible cohort studies which were retrospective in design. These studies contained data on two of the pre-specified outcomes, endometritis and puerperal fever. Other secondary outcomes such as perineal infection and/or any infection, hospital stay duration, sepsis, hemorrhage >1000 ml or hospital readmissions were not reported on excluding puerperal fever.

A meta-analysis showed no significant reduction in the incidence of endometritis (odds ratio [OR] 0.84, 95 % confidence interval [CI] 0.38 to 1.85, three studies, 567 women) and puerperal fever (OR 0.99, 95 % CI 0.38 to 2.27, one study, 302 women).

**Conclusions:**

There is currently no evidence to suggest beneficial effects for routine antibiotic use in women undergoing manual placental removal following vaginal birth. In appropriate settings, further research is required to determine whether a policy of routine antibiotic prophylaxis for the procedure should be maintained or discouraged.

## Background

In the third stage of labor, after delivery of the infant, the placenta spontaneously detaches from the myometrium [1, 2]. When this does not occur, the placenta is said to be ‘retained’. Studies have shown underlying placental and/or uterine abnormalities to be risk factors for a retained placenta [3]. The time frame for diagnosis post-delivery is still ambiguous, however, a 30–60 min time lapse is widely accepted [4, 5]. Existing reviews reported a varying incidence of 1.5–2.7 % in low-resource to high-resource settings respectively, using a 30-min mark-off point [5, 6].

Manual removal of the placenta is indicated if controlled cord traction and the use of uterotonics fails [6–8]. This procedure involves insertion of the hand into the uterus with the aim of separating the placenta from the implantation site, and therefore carries a possible risk of contamination in the uterine cavity. Antibiotic prophylaxis, usually broad spectrum, is routinely administered to reduce infectious morbidities and/or mortalities [9–12]. No evidence exists from randomized control studies or systematic reviews supporting or refuting the practice.

A Cochrane review on the subject initially conducted in 2006 and updated in 2014 did not identify any eligible randomized controlled study [13]. Synthesizing evidence from non-randomized studies is justified in the absence of randomized studies and has been shown to corroborate results from randomized studies regardless of the subject [14]. Hence, we conducted a systematic review on available data from relevant non-randomized studies to determine the efficacy of routine prophylactic antibiotics and if efficacious, the optimal antibiotic regimen for the procedure. This study was conceived as part of the preparation of the evidence base for the World Health Organization (WHO) recommendations for prevention and treatment of maternal peripartum infections.

## Methods

### Search strategy

Based on a pre-specified protocol prepared in line with guidelines in the Cochrane Handbook for Systematic Reviews [14], we conducted a detailed search on January 28, 2015 for eligible studies on MEDLINE, EMBASE, the Cochrane Library and CINAHL databases using specific search terms that included ‘delivery’, ‘obstetric’, ‘placenta’, ‘retained’, ‘anti-infective agents’ and ‘chemoprevention’ (see Appendix S1).

Initially, studies were selected if they were conducted to answer either of these two questions: (1) What are the effects of routine antibiotic prophylaxis on maternal infectious morbidities and mortality, when used for manual removal of the placenta in vaginal deliveries?; and, (2) What is the comparative effectiveness and safety of different antibiotic regimens used for preventing infectious maternal morbidities during manual removal of the placenta? This systematic review was conducted in accordance with the principles of Declaration of Helsinki. Due to the study design, there was no need for ethics approval as the studies were freely available in the public domain.

### Eligibility criteria

All non-randomized studies involving women undergoing manual placental removal after vaginal birth, where the use of antibiotics was compared with no treatment or placebo for prophylaxis against maternal infection. Cluster, quasi-randomized control studies, controlled-before-after studies, cohort and case-control studies were all eligible for inclusion. Comparative studies that reported on comparative use of antibiotic prophylaxis for vaginal births were included while similar studies addressing same comparison but in operative deliveries or a mixed population of operative and vaginal deliveries were excluded, as were studies that contained no data on individual delivery methods.

### Data collection and assessment

For this systematic review, the Preferred Reporting Items for Systematic Reviews and Meta-Analyses [15] (PRISMA) method of reporting was used.

The titles and abstracts of all resulting citations from search strategy were examined independently for eligibility by two review authors (CEC and AJQP) irrespective of the publication status (published and unpublished) or language of publication.

After the removal of duplicates, selected studies were then retrieved and further evaluated for inclusion on the basis of full-text appraisal. Disagreements were resolved by discussions with the third review author (EO). Data extraction using a standardized form was performed independently by two review authors (CEC and AJQP), which included information on the study design, facilities, participants, settings and outcomes.

Pre-specified outcomes included infectious morbidities common to obstetric procedures. [5, 16]. Primary outcome was postpartum endometritis as defined by the authors and secondary outcomes included puerperal fever defined as temperature of 38.0 °C or higher, perineal infections, hospital stay duration, sepsis, postpartum hemorrhage, hospital readmission, drug side effects, and neonatal-related outcomes (jaundice, sepsis, intensive care unit admission).

Rating the methodological quality of eligible studies was performed independently by two review authors (CEC and AJQP) using the Newcastle Ottawa scale [17], which involved assessing the methods of participant selection, comparability and outcomes among eligible studies. The quality of the evidence obtained from included studies were further assessed using the Grading of Recommendations Assessment, Development and Evaluation (GRADE) approach [18–20].

### Data analysis/synthesis

Data from included studies (Table 1) was pooled and meta-analysis conducted using a random effects model. Meta-analysis was performed using Revman 5.3 according to the meta-analysis of observational studies in epidemiology (MOOSE) guidelines.

**Table 1:** Characteristics of included studies

Author, year	Study design	Total study population	N	Enrolment period	Location	Type of obstetric procedure	Available outcomes
			(treatment, control)				
Katsulov 1983 [21]	Retrospective cohort study	100	30/70	1976–1977	Bulgaria	Women who underwent manual placenta removal	Endometritis
Von Rechlin et al. 1988 [22]	Retrospective cohort study	302	65/237	An undefined period of 6 years	Germany	Women undergoing manual placental removal	Endometritis, fever greater than 37.5 °C
Tandberg et al. 1999 [23]	Retrospective cohort study	24,750 births	61/104	1990–1994	Norway	Women who underwent a manual placenta removal	Endometritis

A Mantel Haenszel model for sparse dichotomous data was applied, and adjusted using the random-effects model and presented as odds ratios with a 95 % confidence interval (CI), a *p*-value of 0.05 and heterogeneity estimates of Tau^2^ and I^2^.

## Results

**Fig. 1:**
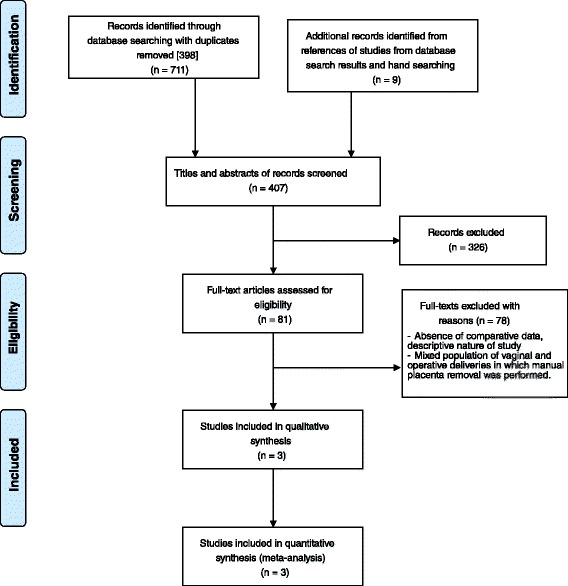
Flow diagram of search results and study selection

The titles and abstracts of 407 studies that were identified from the keyword search of relevant databases after elimination of duplicates were independently assessed by two review authors (CEC and AJQP). After initial screening, the full texts of 81 potentially eligible studies were retrieved for further appraisal (Fig. 1). A sizeable number of studies on the subject were published in English and other foreign languages, however, these studies merged data for both vaginal and operative deliveries and were therefore ineligible for inclusion. Only three studies met the inclusion criteria.

### Effects of antibiotic prophylaxis on infectious maternal morbidities in vaginal deliveries involving manual placenta removal

All three eligible studies were retrospective in design and conducted in hospitals in Norway (1999), Bulgaria (1983) and Germany (1988) [21–23]. The study population and intervention were consistent across studies. Antibiotics for prophylaxis were introduced for manual placenta removal after placenta retention was diagnosed and women with a prior history of fever were ineligible for inclusion in the study population. All three studies reported on the pre-specified primary outcome, postpartum endometritis. All secondary outcomes (perineal infection and/or any infection, hospital stay duration, sepsis, hemorrhage >1000 ml, hospital readmissions) were not reported on excluding puerperal fever. Only one of the eligible studies reported on this.

Two studies reported on the antibiotic agent used. Von Rechlin et al. [22] used Mebacid/Sulfamerazine (at least over 10 days, 1 g per 1 day and then 0.5 g daily), while Katsulov [21] used 2 g cefamandole (cephalosporin family) for an unstated short duration. In the third study [23], the antibiotic prophylaxis agent was unnamed and the dosage was not given. The authors reported routine use of antibiotics in the center, with the practice maintained throughout the study period.

Both studies loosely defined endometritis as fever greater than 37.5 °C in addition to clinical evidence (not clearly stated in either paper, though probably based on clinician’s judgment) and puerperal fever was defined as a persistent or uninterrupted temperature record of 37.5 °C.

The third study was published in English and better defined endometritis as a temperature of 38.0 °C, excessive uterine tenderness or elevated C-reactive protein (±50 mg/l).

Von Rechlin et al. [22] reported a comparable incidence of fever in both the prophylaxis group (89.2 %) and the control group (89.9 %). The other two studies [21, 22] reported only on endometritis as an outcome. Both studies showed no difference between the intervention and control group. Katsulov [21] showed no incidence of endometritis in the prophylaxis group (0 %) compared to control (10.1 %), although this difference was not significant. Tandberg et al. [23] detected a similar incidence of endometritis in both the prophylaxis (1.6 %) and control (1.9 %) groups.

Follow-up periods ranged from the period of administration of antibiotics (6 days) to the 1st month postpartum, although the period was unstated in Von Rechlin et al. [22].

A meta-analysis similarly showed no difference between prophylaxis and non-prophylaxis groups. For puerperal fever, no difference was observed between groups (odds ratio (OR) 0.93, 95 % confidence interval (CI) 0.38 to 2.27, one study, 302 women).

**Fig. 2:**
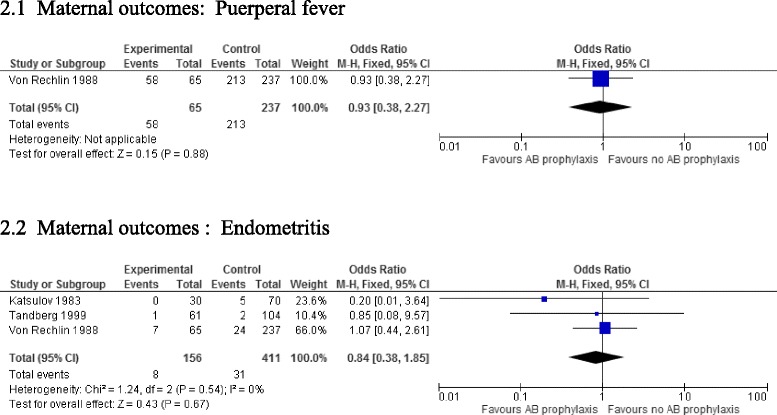
Maternal outcomes: **a** Puerperal fever, **b** Endometritis. Adjusted odds ratio for antibiotic prophylaxis versus no prophylaxis in manual placenta removal procedures during vaginal delivery

Similarly, incidence of endometritis was similar in both groups (OR 0.84, 95 % CI 0.38 to 1.85, three studies, 567 women) (Fig. 2). No adverse effects or other secondary outcomes were reported, and no heterogeneity was observed across studies.

Assessment of eligible studies using the Newcastle Ottawa Scale generally showed a low quality of evidence (Table 2), and both outcomes (endometritis and fever) were judged very low in overall quality using the GRADE tool (Table 3).

**Table 2:** Newcastle-Ottawa Scale (NOS) risk of bias assessment for included studies

NOS (Newcastle-Ottawa Scale) risk of bias assessment for included studies			
Study ID	Von Rechlin et al. 1988 [22]	Tandberg et al. 1999 [23]	Katsulov 1983 [21]
Representativeness of exposed cohort - Truly representative of the average woman (*) - Somewhat representative of the average woman (*) - Selected group of users -No description of the derivation of the cohort			
Selection of non-exposed cohort - Drawn from the same community as the exposed cohort (*) - Drawn from a different source - No description of the derivation of the non-exposed cohort			
Ascertainment of exposure - Secure records (e.g., surgical records) (*) - Structured interview (*) - Written self-report - No description			
Demonstration that outcome of interest was not present at the start of the study - Yes (*) - No		*	
Comparability of cohorts on the basis of the design or analysis - Study controls for gestational age and/or birth weight (*) - Study controls for any additional factor (*)		*	
Assessment of outcome -Independent blind assessment (*) - Record linkage (*) -Self-report -No description		*	*
Follow-up long enough for outcomes to occur - Yes (*) - No	*	*	*
Adequacy of follow-up of cohorts - Complete follow-up – all subjects accounted for (*) - Subjects lost to follow-up unlikely to introduce bias, or description provided of those lost (*) - No statement	*	*	*
Total number of stars	2	5	3

**Table 3:** GRADE tables (Non-randomized studies)

Quality assessment							No of patients		Effect		Quality	Importance
No. of studies	Design	Risk of bias	Inconsistency	Indirectness	Imprecision	Other considerations	Ab prophylaxis versus none	Control	Relative (95 % CI)	Absolute		
Puerperal fever												
1	observational studies	very serious^a^	no serious inconsistency	no serious indirectness	serious^b^	none	58/65 (89.2 %)	213/237 (89.9 %)	OR 0.93 (0.38 to 2.27)	7 fewer per 1000 (from 127 fewer to 54 more)	⊕ΟΟΟ VERY LOW	CRITICAL
								89.9 %		7 fewer per 1000 (from 127 fewer to 54 more)		
Endometritis												
3	observational studies	very serious^a^	no serious inconsistency	no serious indirectness	serious^b^	none	8/156 (5.1 %)	31/411 (7.5 %)	OR 0.84 (0.38 to 1.85)	11 fewer per 1000 (from 45 fewer to 56 more)	⊕ΟΟΟ VERY LOW	CRITICAL
								7.1 %		11 fewer per 1000 (from 43 fewer to 53 more)		

Question: Antibiotic prophylaxis versus none for manual placental delivery during vaginal birth

Settings: Hospitals in Germany, Norway and Bulgaria

^a^Study contributing data had design limitations based on Newcastle Ottawa Scale rating, non-randomized data collection

^b^Wide confidence interval crossing the line of no effect

### Prophylactic antibiotic regimen for reduction of maternal infectious morbidities during manual placenta removal in vaginal birth

No eligible studies were found comparing antibiotic classes or regimens for prophylaxis in vaginal deliveries involving manual placenta removal.

## Discussion

### Main findings

No significant benefit was observed for the routine use of prophylactic antibiotics on the reduction of maternal febrile morbidities or postpartum endometritis for manual placenta removal. However, the low quality of evidence limits definite conclusions.

Included studies were comparable in terms of study population, intervention and outcome. All three studies individually showed no difference between the control and experimental groups for both fever and endometritis outcomes.

Meta-analysis further showed a similar incidence of reported maternal morbidities (fever and endometritis) between groups. In the random effects analysis, the Von Rechlin study weighted 81 % of the pooled weight and appears to favor the use of antibiotic prophylaxis compared to no prophylaxis, though this should be interpreted with caution due to the study’s small sample size and low quality of evidence.

### Strengths and limitations

No evidence exists to date that assesses routine antibiotic prophylaxis for manual placental removal in vaginal birth. This systematic review provides comparable alternative evidence to currently lacking randomized controlled studies and may represent the only systematic evidence available on the subject.

The detailed search strategy, methodology, statistical analyses, absence of statistical heterogeneity and language restrictions, however, lends credence to this review.

Inclusion of studies published in languages other than English ensured the inclusion of important studies on the subject.

This review was limited to evidence from a small number of low quality non-randomized studies and few outcomes due to a clear absence of randomized studies on the subject. Three studies met the inclusion criteria and reported on only two outcomes. Collated data was from observational studies conducted in two developed countries (Germany and Norway) and one developing country (Bulgaria) and may not be globally representative or applicable as policies on antibiotic administration may vary across income settings. The lack of heterogeneity across studies further adds to the validity of the study.

Routine antibiotic prophylaxis is increasingly associated with, antibiotic resistance [24]. Its administration needs to be assessed in the light of emerging resistance and inherent benefits to justify and balance recommendations for routine use; however, eligible studies included in this review failed to report on these points.

In addition, no reports were found on other outcomes in any of the eligible studies or any data comparing the use of different types of prophylactic antibiotics for manual placenta removal in vaginal deliveries. This restricted our evaluation of the effectiveness of routine antibiotic prophylaxis or the definition of an optimal regimen. Studies aiming to determine optimal doses may prove helpful in the future.

## Conclusion

Available evidence is insufficient to support routine antibiotic prophylaxis for manual placental removal following vaginal birth. This study highlights the need for robust randomized control studies in both low- and high-income settings that will incorporate relevant outcomes such as adverse effects of prophylaxis. Knowledge of this may help inform better assessment of the efficacy of routine antibiotic prophylaxis in obstetric vaginal procedures. The decision to administer antibiotics for manual placenta removal should be selective and based on a balance between the inherent value to the patient and the clinician experience in the light of increasing antibiotic resistance.

Increase in antibiotic resistance in recent years highlights the need for selective prophylaxis to be integrated into best practice and policy against a background of existing area-specific norms and policies.

Successful prevention of maternal infectious morbidities and mortalities resulting from manual removal of the placenta in vaginal births may prove invaluable towards maternal health improvement.

## Abbreviations

MEDLINE: Medical Literature Analysis and Retrieval System Online
EMBASE: Excerpta MEdica Database
CINAHL: Cumulative Index of Nursing and Allied Health Literature
CI: Confidence Interval
NOS: NewCastle-Ottawa Scale
WHO: World Health Organisation
OR: Odds Ratio
PRISMA: Preferred Reporting Items for Systematic Reviews and Meta-analyses GRADE, Grades of Recommendation, Assessment, Development and Evaluation
MOOSE: Meta-analysis of Observational Studies in Epidemiology
